# European Bat Lyssavirus Transmission among Cats, Europe

**DOI:** 10.3201/eid1502.080637

**Published:** 2009-02

**Authors:** Laurent Dacheux, Florence Larrous, Alexandra Mailles, Didier Boisseleau, Olivier Delmas, Charlotte Biron, Christiane Bouchier, Isabelle Capek, Michel Muller, Frédéric Ilari, Tanguy Lefranc, François Raffi, Maryvonne Goudal, Hervé Bourhy

**Affiliations:** Institut Pasteur, Paris, France (L. Dacheux, F. Larrous, O. Delmas, C. Bouchier, M. Goudal, H. Bourhy); Institut de Veille Sanitaire, Saint-Maurice, France (A. Mailles, I. Capek); Direction Départementale des Services Vétérinaires de la Vendée, La Roche-sur-Yon, France (D. Boisseleau); Centre Hospitalier Universitaire de Nantes, Nantes, France (C. Biron, F. Raffi); Clinique Vétérinaire du Bas-Poitou, Fontenay-le-Comte, France (M. Muller); Clinique Vétérinaire Roosevelt, Vannes, France (F. Ilari, T. Lefranc)

**Keywords:** Rabies, lyssavirus, chiroptera, cats, diagnosis, spillover, transmission, Europe, dispatch

## Abstract

We identified 2 cases of European bat lyssavirus subtype 1 transmission to domestic carnivores (cats) in France. Bat-to-cat transmission is suspected. Low amounts of virus antigen in cat brain made diagnosis difficult.

Most countries in western Europe are currently free of rabies in terrestrial mammals, as was the case in France during 2001–2008 (*1*). However, rabies still remains a public health problem in these countries because of natural circulation of bat-specific viruses (order Mononegavirales, family *Rhabdoviridae*, genus *Lyssavirus*) such as European bat lyssaviruses (EBLVs). These viruses are divided into genotypes 5 (EBLV-1) and 6 (EBLV-2); the first genotype is subdivided into subtypes a and b (*2*). Knowledge of the prevalence and epidemiology of EBLV is limited (*2*–*5*). To date, natural transmission of EBLV-1 has been reported in a limited number of terrestrial mammals, including 5 sheep in Denmark (*6*) and 1 stone marten in Germany (*7*) (Table 1). Since 1985, only 3 human deaths from EBLVs have been confirmed (*3*) (Table 1). We describe 2 documented cases of spillover transmission of EBLV in domestic carnivores (cats, *Felis domesticus*) in Europe.

**Table 1:** Confirmed cases of EBLV spillover transmission to terrestrial mammals and humans, Europe*

Host (no. cases)	Year of isolation	Location	Clinical signs or disease	Techniques used for rabies diagnosis on original brain samples					EBLV type
				FAT	RTCIT	MIT	ELISA	RT-PCR	
Sheep† (4)	1998	Western Jutland, Denmark	Neurologic disorders	+ (weak)	+ (1 of 4 sheep)	–	ND	+ (only 1 sheep tested)	1a
Stone marten‡ (1)	2001	Burg, Saxony-Anhalt, Germany	No obvious clinical signs	– (repeated testing)	+ (weak)	+	ND	+	1a
Sheep† (1)	2002	Western Jutland, Denmark	Neurologic disorders	+	+	–	ND	ND	1a
Domestic cat§ (1), cat no. 1	2003	Vannes, Morbihan, France	Emaciated, moderate dehydration, FIV detected	– (repeated testing)	– (after 3 cell culture passages)	–	+	+	1b
Domestic cat§ (1), cat no. 2	2007	Fontenay-le-Comte, Vendée, France	Neurologic disorders, aggressive	+ (weak)	+ (weak after 2 cell culture passages)	+	+ (variable)	+	1a
Man, 30 y of age (1)	1985	Helsinki, Finland	Rabies	+	+	+	ND	ND	2
Girl, 11 y of age (1)	1985	Belgorod, Russia	Rabies	–	ND	+	ND	ND	1a
Man, 55 y of age (1)	2002	Angus, Scotland	Rabies	+	+	+	ND	+	2

*EBLV, European bat lyssavirus; FAT, direct immunofluorescence antibody test; RTCIT, rabies tissue culture infection test; MIT, mouse inoculation test; RT-PCR, reverse transcription–PCR; ND, not determined; FIV, feline immunodeficiency virus. †*Ovis aries*. ‡*Martes foina*. §*Felis domesticus*.

## The Study

In November 2003, a 6-month-old female stray cat (cat no. 1) was found ill in a public garden in Vannes (Morbihan District) in western France and taken to a veterinary clinic. This animal had convulsions and moderate dehydration and was emaciated. It was infected by feline immunodeficiency virus, which was compatible with the clinical symptoms. The veterinarian was bitten while providing veterinary care to the cat. After a few days, the cat recovered and was impounded for veterinary surveillance. It died suddenly the following night. No information about potential contact with bats was available.

On November 8, 2007, an 18-month-old female cat (cat no. 2) was taken by its owner to a veterinarian in Fontenay-le-Comte (Vendée District) in western France because of abnormal behavior. The owner reported having been bitten by the cat. The next day, the cat showed severe central neurologic disorders and aggressive behavior. It died during the next night. Its outdoor access appeared to have been restricted. Two months later, the carcass of a bat (*Eptesicus serotinus*) was recovered in the same area of Fontenay-le-Comte and submitted for rabies testing.

Recommended techniques for rabies diagnosis were used for all animals (*8*). For cat no. 1, results of a repeated direct immunofluorescence antibody test (FAT) with a polyclonal antirabies conjugate (Bio-Rad, Marnes-la-Coquette, France) performed on different cortex and spinal bulb smears were negative. Viral isolation by using a rabies tissue culture infection test (RTCIT) was also unsuccessful, as was attempted isolation of virus by using a mouse inoculation test (MIT) (Table 1). The only test routinely used that gave a positive result was an antigen-capture ELISA (WELYSSA) for lyssavirus antigen (*9*). The presence of EBLV RNA (03011FRA) was determined by reverse transcription–PCR (RT-PCR) targeting short viral gene regions (*5*).

Lyssavirus antigens were repeatedly detected by FAT in different areas of the brain of cat no. 2. Viral isolation by using RTCIT was positive only after the second cell culture passage. Results for isolation of EBLV (07240FRA) by MIT were positive. Lyssavirus antigen detection by WELYSSA was variable, depending on the part of the brain tested. Viral RNA was detected by RT-PCR (Table 1). The bat was positive for EBLV by FAT, RTCIT (08120FRA), MIT, and RT-PCR.

**Figure 1:**
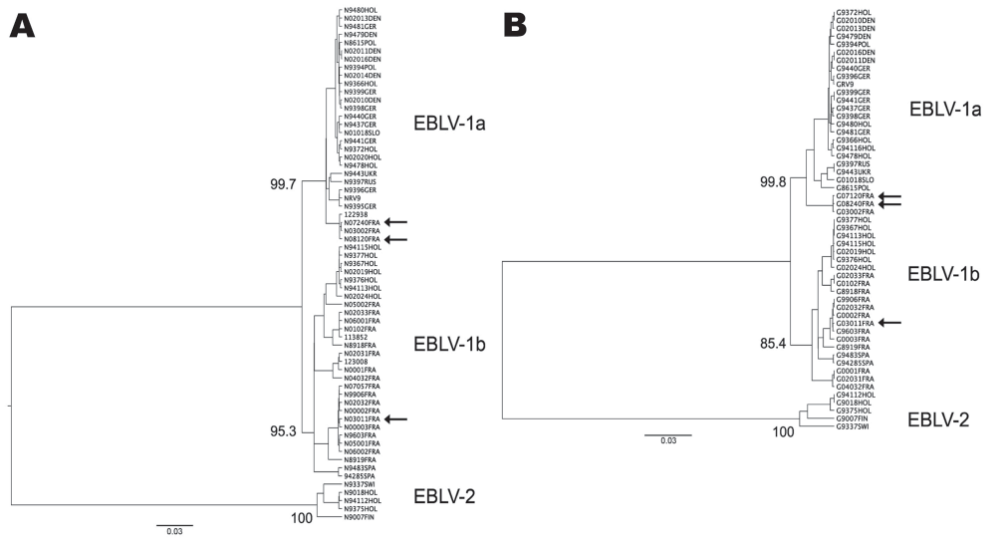
Phylogenetic tree comparing nucleotide sequences of A) nucleoprotein (372 nt, position 63 from the translation initiation site) and B) glycoprotein (547 nt, position 640 from the translation initiation site) genes of spillover transmission of European bat lyssavirus-1 (EBLV-1) in terrestrial mammals and human with representative isolates of the diversity of EBLV-1 in Europe. Cases described in this report are indicated by the arrows. For each dataset, we inferred a maximum clade credibility phylogenetic tree by using the Bayesian Markov Chain Monte Carlo method available in the Bayesian Evolutionary Analysis Sampling Trees software (http://beast.bio.ed.ac.uk). This analysis used a relaxed (uncorrelated lognormal) molecular clock and the HKY85 + Γ_4_ model of nucleotide substitution. All horizontal branches are scaled according to the number of substitutions per site. Bootstrap values are indicated at the nodes. All GenBank accession numbers corresponding to full-length or partial nucleoprotein and glycoprotein nucleotide sequences were previously described (*2*,*10*) except for nucleoprotein nucleotide sequences of isolates 03011FRA (EU636795), 04032FRA (EU636794), 05001FRA (EU636790), 05002FRA (EU636789), 06001FRA (EU636791), 06002FRA (EU636792), and 070057FRA (EU636793) and glycoprotein nucleotide sequences of isolates 03011FRA (EU636787) and 04032FRA (EU636788).

Nucleotide sequencing and phylogenetic analysis identified isolate 03011FRA as EBLV-1b and isolates 08120FRA and 07240FRA as EBLV-1a (Figure 1). Sequencing of the complete genome (*10*) of the 2 EBLV-1a isolates showed a high percentage of homology (Table 2).

**Table 2:** Percentage nucleotide divergence between EBLV-1a strains isolated from a cat (07240FRA) and bats (08120FRA, 03002FRA, and RV9)*

Gene	Sequence	Strain		
		07240FRA/08120FRA	07240FRA/03002FRA	07240FRA/RV9
Complete genome	nt	0.1 (12)	0.4 (36)	2 (226)
Nucleoprotein	nt	0	0.2 (2)	1.5 (19)
	aa	0	0	0
Phosphoprotein	nt	0.1 (1)	0.4 (4)	2.3 (23)
	aa	0.3 (1), **I149T**	0.7 (2), **I149T**, **G175D**	1.7 (7), **Q147R**, **I149T**, **T156A**, **F169S**, **P174L**, **G175D**, **G266S**
Matrix	nt	0	0.3 (2)	1.5 (7)
	aa	0	0	1 (2), **N2K**, **I155M**
Glycoprotein	nt	0	0.2 (3)	2.1 (32)
	aa	0	0.2 (1), **S489P**	0.8 (4), **L244Q**, **S278N**, **S489P**, **A521T**
Polymerase	nt	0.03 (2)	0.3 (16)	1.9 (109)
	aa	0	0.2 (4), **A97T**, **G1160D**, **T1754I**, **R1894S**	0.3 (5), **R315K**, **I391V**, **K980R**, **T1754I**, **R1894S**

*Strains 07240FRA and 08120FRA were isolated from cat no. 2 and from a bat (*Eptesicus serotinus*) found dead in the same area (Fontenay-le-Comte, France), respectively. Strain 03002FRA was isolated from another bat (*E. serotinus*) collected ≈100 km from Fontenay-le-Comte in 2003 (*10*). Isolate RV9 was collected from a bat (*E. serotinus*) in 1968 in Germany (*15*). Numbers of substitutions are indicated in parentheses. Type of amino acid substitutions are indicated in **boldface**. GenBank accession numbers for full-length genomic sequences corresponding to strains 07240FRA, 08120FRA, 03002FRA, and RV9 are EU626552, EU626551, EU293109, and EF157976, respectively. EBLV, European bat lyssavirus; nt, nucleotide; aa, amino acid.

After identification of these 2 cases of spillover transmission of EBLV-1 to domestic cats, postexposure prophylaxis measures were implemented. The veterinarian who was bitten by cat no. 1 received a booster rabies vaccination, and 15 persons exposed to cat no. 2 during the 2-week critical period before its death received appropriate postexposure treatment on the basis of national and international recommendations of the World Health Organization (*8*,*11*). Two family dogs potentially exposed to cat no. 2 and previously vaccinated received a booster vaccination. Cross-neutralization data obtained with human serum samples and in rodent models suggest that preexposure and postexposure treatments for rabies are effective against EBLV-1.

Control measures were implemented to prevent potential further contaminations, although cats represent naturally dead-end host for rabies (and for lyssavirus), thereby limiting any risk for transmission to other mammals. City authorities conducted a census of all domestic animals in the neighborhood where cat no. 2 lived. All cats, dogs, and ferrets were identified by microchips and kept under veterinary surveillance. Dogs had to be leashed and cats kept indoors during the next 2 months.

## Conclusions

We report 2 documented cases of natural infection of domestic cats by EBLV-1 lyssaviruses presently circulating in European bats (*2*–*5*). Our study demonstrates that subtypes EBLV-1a and EBLV-1b can cross the species barrier, although cat no. 1 probably died of feline leukemia. However, cat no. 2 died with neurologic signs compatible with rabies and was positive for EBLV by FAT, the reference technique. Direct transmission of EBLV-1 from bats to cats seems the most realistic explanation for these cases because cats prey on bats and have numerous contacts with them (*3*,*12*,*13*). Identification of a highly homologous EBLV-1a isolate from a rabid bat found in the same location as cat no. 2 supports this hypothesis.

Difficulties in EBLV-1 detection in the brain of these 2 cases of spillover transmission are reminiscent of transmission previously reported (*6*,*7*) (Table 1), potentially caused by a low amount of virus antigen in the brain. This finding further underlines the importance of using different techniques to diagnose rabies caused by EBLVs (*9*). This issue raises questions about the true incidence of these viruses among cats.

**Figure 2:**
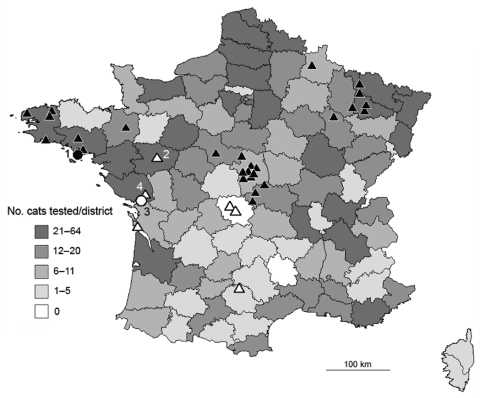
Distribution of cats analyzed during 2004–2007 and of bats found positive for European bat lyssavirus (EBLV) in France during 1989–2007. Distribution of 1,506 cats tested during 2004–2007 by direct immunofluorescence antibody test, rabies tissue culture infection test, and an antigen-capture ELISA is given by district. Precise location of the 2 infected index (positive) cats and positive bats (n = 32) are indicated by circles and triangles, respectively, and associated with numbers 1, 2, 3, and 4 for isolates 03011FRA, 03002FRA, 07240FRA, and 08120FRA, respectively. EBLV-1a and EBLV-1b isolates are indicated in black and white, respectively. Map was constructed by using Articque’s C&D software (www.articque.com) and published according to Articque's publication policy.

At the Institut Pasteur during 1997–2007, a total of 6,097 cats suspected of having rabies and originating from all districts in France showed negative results for rabies by 2 recommended techniques (FAT and RTCIT). Among them, all animals tested since 2004 (1,506 cats), except cats no. 1 and no. 2, were also negative for rabies by WELYSSA (Figure 2), which suggests that transmission of EBLVs from bats to cats, although possible, is rare. Furthermore, terrestrial mammals seem to represent dead-end hosts for EBLVs, as suggested by results of experimental EBLV inoculations in several mammals such as cats, dogs, ferrets, mice, red foxes, or sheep (*6*,*7*,*14*). These animals are susceptible to infection with EBLVs but seem unlikely to actively transmit EBLVs to a new host.

Comparative analysis of the full-length genomic sequence of the EBLV-1a from cat no. 2 isolate 07240FRA with bat isolate 08120FRA and with another bat (*E*. *serotinus*) isolate (03002FRA) collected in 2003 ≈100 km from Fontenay-le-Comte showed high similarity (Table 2). This finding indicates that heterologous passage of EBLV-1a in a cat did not select mutants. Similarly, the lower similarity observed with a genomic sequence collected from a bat RV9 (*15*) (*E*. *serotinus*) in 1968 in Germany also indicates that EBLV-1 evolution is shaped by slow genetic drift (*2*).

No secondary cases originating from cat no. 1 and cat no. 2 were reported (after 6 months of follow-up for cat no. 2). However, improving surveillance and raising awareness to better understand the epidemiology of lyssaviruses are necessary. Persons bitten by bats or by any carnivorous animal are advised to wash wounds with water and soap and to seek medical attention (*8*,*11*).
